# The first complete mitochondrial genome of *Adelges tsugae* Annand (Hemiptera: Adelgidae)

**DOI:** 10.1080/23802359.2020.1772682

**Published:** 2020-06-02

**Authors:** Hsin-Ting Yeh, Chiun-Cheng Ko, Li-Wei Wu

**Affiliations:** a The Experimental Forest, National Taiwan University, Nantou County, Taiwan; b Department of Entomology, National Taiwan University, Taipei, Taiwan

**Keywords:** Invasive insect, spruce, galling aphid, forest pest

## Abstract

Hemlock wooly adelgid (HWA), *Adelgests ugae* Annand (Hemiptera: Adelgidae), is a species native to Asia but later ravages Endangered hemlock forests (*Tsuga* spp.) in eastern North America. In this study, we obtained the first complete mitochondrial genome of HWA (16,509 bp in length) using meta-genomic sequencing method. The HWA mitogenome has a general gene annotation as other aphids, comprising 13 protein-coding genes, 22 transfer RNAs, and 2 ribosomal RNAs. Our phylogenetic result showed Aphidoidea is sister to Coccoidea and the newly sequenced mitogenome is put on the correct position, sister to *Adelgeslaricis*.

Hemlock wooly adelgid (HWA), *Adelgest sugae* Annand, is a species native to Asia, but has caused the collapse of the native hemlock (*Tsuga* spp.) forest ecosystem in eastern North America (Orwig and Foster 1998; Havill et al. 2016; Limbu et al. 2018). Like other aphid relatives, HWA has host alternation and complex polymorphism in the lifecycles (Sano and Ozaki 2012; Limbu et al. 2018). In the past, the polymorphism exhibited during the host alternation process makes the identification of aphid species difficult and in recent times, the development of molecular technologies has provided a solution (Stern et al. 1997; Foottit et al. 2009; von Dohlen 2009). HWA is a native aphid species in Taiwan and the secondary host is *T. chinensis* var. *formosana*, which is distributed in the high mountains of Taiwan about 2100–3000 meters (Takahashi 1937). Morrison spruce, *Piceamorrisonicola*, is the primary host of HWA in Taiwan, and HWA will induce a small cone-shaped gall on the branch.

Typically, Aphidoidea (belongs to order Hemiptera) divided into three families, Adelgidae, Aphididae, and Phylloxeridae, while HWA belongs to the family Adelgidae (Blackman and Eastop 1994, 2006; Remaudière and Remaudière 1997; Favret et al. 2015). In recent years, there have been many reports of complete mitochondrial sequences of aphids, but the species are mainly aphids of the Aphididae (Thao et al. 2004; The International Aphid Genomics Consortium, 2010; Wang et al. 2013, 2014; Zhang et al. 2014; Wang et al. 2015, 2016; Ren et al. 2016; Zhang, Zheng, et al. 2016a; Zhang, Luo 2016b; Li et al. 2017; Song et al. 2019; Wei et al. 2019; Zhang et al. 2019; Nong et al. 2020; Voronova et al. 2020). This report has presented the first complete mitochondrial sequence of the Adelgidae.

In May, 2016, the HWA were collected from their hostplant, *T. chinensis* var. *formosana*at the middle-mountainous region of central Taiwan (Meifong, Nantou County, 24°05′15.9″N, 121°10′29.5″E). A colony of the specimens was deposited in the Insect Collections of National Museum of Natural Science (collection number:NMNS ENT 8207-1), Taichung, Taiwan, but five of them were token and extracted their genomic DNAs for next-generation sequencing byMiseq platform. Total 6,403,862 reads (average trimmed length, 213.3 bp) were obtained after removing low DNA quality regions (below Q20) using CLC Genomics Workbench 9 (CLC bio, Aarhus, Denmark). The trimmed reads were *de novo* assembled into contigs with the setting of 97% sequence similarity via software CLC Genomics Workbench and megahit (Li et al. 2015). The mitogenome-like sequences were filtered out by comparing to a Hemiptera reference, which contains 226 related mitogenomic sequences. The assembled contigs were combined and edited to generate mitogenomic sequences using Sequencher 4.10 (GeneCode, Boston, USA). Then, the complete mitogenome of HWA is obtained (Accession number MT263947), 16,059 bp in length. Gene regions and order were predicted using MITOS2 webserver (Bernt et al. 2013) and the gene positions were double checked with the public sequences of *Adelgeslaricis* (KP722589), *Diuraphisnoxia* (NC_022727), and *Diaphorinacitri* (NC_030214). The newly sequenced HWA mitogenome shows the same gene order as the reference, *Diuraphisnoxia*, comprising 13 protein-coding genes, 22 transfer RNAs, and 2 ribosomal RNAs.

To investigate phylogenetic position of HWA, 27 related species were sampled from NCBI to infer Aphid phylogenetic tree (Figure 1). Maximum likelihood (ML) method (Stamatakis 2006; Ott et al. 2007) was inferred based on 37 mitochondrial genes. The substitution model was set to GTRGAMMA and the partition scheme was set as gene partition, except for 22 tRNAs, which were concatenated as one partition. Nodal supports were examined using 1000 bootstraps with 10 additional ML searches to improve bootstrapping (Ott et al. 2007). The phylogenetic relationship shows that Aleyrodidae is the sister to the clade including Coccoidea and Aphidoidea and the newly sequenced mitogenome, *Adelgests nugae*, is sister to *Adelgeslaricis*.

**Figure 1.**
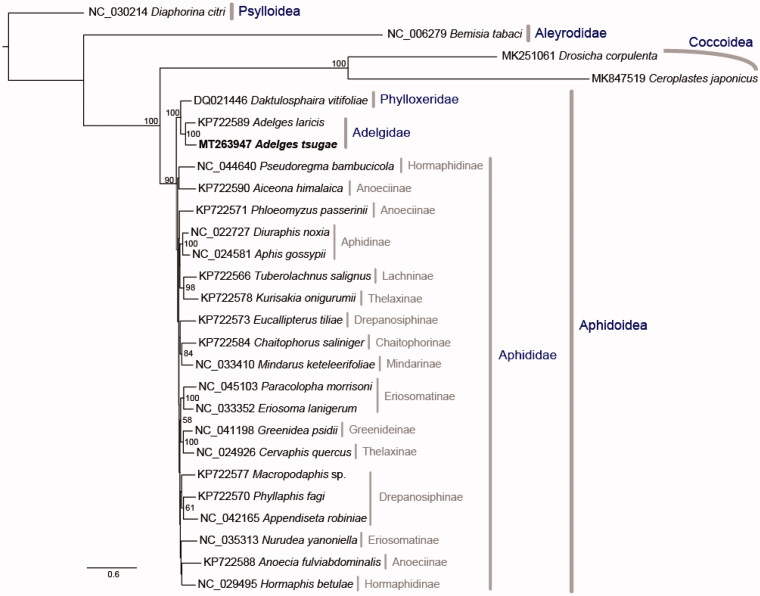
The ML phylogeny of the Aphidoidea, Psylloidea was set as an outgroup and the total aligned length (37 genes) is 15,167 bp.

In Virginia, U.S., HWA was first reported in the 1950s and it has now become the most serious pest of native hemlock forests in eastern North America (Limbu et al. 2018). It is speculated that the HWA populations of eastern North America are related to the populations from southern Japan based on molecular data (Havill et al. 2006, 2016). Havill et al. (2016) set the eight monophyletic lineages of HWA based on 748 individuals among 133 sampling sites, but phylogenetic relationships among some of these lineages are still ambiguous. Therefore, our newly produced mitogenome could be used not only to compare with other HWA lineages but also to be a reference to infer phylogenetic relationships within Aphidoidea.

## Data Availability

The HWA slide specimens supporting this study were deposited in the Insect Collections of National Museum of Natural Science (collection number:NMNS ENT 8207-1), Taichung, Taiwan, andthe complete HWA mitogenome is available in GenBank (Accession number MT263947; live link: https://www.ncbi.nlm.nih.gov/nuccore/MT263947).
